# What makes a competent clinical teacher?

**Published:** 2012-09-30

**Authors:** Stephen Wealthall, Marcus Henning

**Affiliations:** 1 Medical Education Development Office, University of Auckland, New Zealand; 2 Centre for Medical and Health Sciences Education, University of Auckland, New Zealand

## Abstract

**Background:**

Clinical teaching competency is a professional necessity ensuring that clinicians’ knowledge, skills and attitudes are effectively transmitted from experts to novices. The aim of this paper is to consider how clinical skills are transmitted from a historical and reflective perspective and to link these ideas with student and teacher perceptions of competence in clinical teaching.

**Methods:**

The reflections are informed by a Delphi process and professional development survey designed to capture students’ and clinicians’ ideas about the attributes of a competent clinical teacher. In addition, the survey process obtained information on the importance and ‘teachability’ of these characteristics.

**Results:**

Four key characteristics of the competent teacher emerged from the Delphi process: clinically competent, efficient organizer, group communicator and person–centred. In a subsequent survey, students were found to be more optimistic about the ‘teachability’ of these characteristics than clinicians and scored the attribute of person-centredness higher than clinicians. Clinicians, on the other hand, ascribed higher levels of importance to clinical competency, efficient organization and group communication than students.

**Conclusions:**

The Delphi process created a non-threatening system for gathering student and clinician expectations of teachers and created a foundation for developing methods for evaluating clinical competency. This provided insights into differences between teachers’ and students’ expectations, their importance, and professional development.

## Background

Clinical teaching has always involved a special relationship between student and teacher which derives from its historical apprenticeship model. As well as absorbing factual information and learning behavioural and psychomotor skills which the teacher checks, the student sees and forms judgements on the clinician’s emotional interaction with patients. Students, both consciously and unconsciously, then use clinician’s interactions with patients as both positive and negative role models, which may eventually shape their own way of interacting with patients and managing their conditions. Because teachers consciously and unconsciously shape students on the journey to becoming clinicians, clinical teachers develop insights into how their clinical skills are transmitted to students. In this personal view paper, we consider how clinical skills are transmitted from a historical and reflective perspective and link these ideas with student and teacher perceptions of competence in clinical teaching.

At present clinical teaching is under pressure in many parts of the world due to increases in the number of students in health profession and organizational and financial pressures requiring clinicians to maximise their clinical throughput. These influences bring pressure on health professional teaching establishments to change traditional small group or individual clinical teaching to other more high-volume formats, thus reducing student opportunity to be directly exposed to real clinician-patient interaction.^1^ The special relationship between clinician-teacher and student was highlighted over 100 years ago by William Osler:^2^ “Medicine is learned by the bedside and not in the classroom.” Osler’s insights into good clinical teaching included patient-centeredness, teaching at the bedside, emphasising careful observation and listening skills, keeping abreast with the latest developments in medical science, and working hard to attain professional excellence – all attributes largely influenced by role modelling. In our view, and consistent with Osler’s seminal ideas,^2^ individual or small group clinical teaching exposure produces graduates who are superior to those trained in the lecture theatre or the many varieties of teaching laboratory. In addition, we believe that the tension between the pressures for clinical efficiency versus clinical student mentoring needs to be resolved by identifying what both students and teachers perceive as good clinical teaching. If such characteristics can be identified we must ensure that clinical teaching concentrates on those essentials.

In the 1970’s, Irby^3^ determined characteristics of good clinical teaching comparatively: “… the major difference between best and worst clinical teachers appears to be the instructional skills of the best (that is, organization and clarity of presentation, enthusiasm, and interaction skills) and the personal attributes of the worst (that is, arrogance, lack of self-confidence, dogmatism, and insensitivity....” This seminal study established the significance of interpersonal skills in good clinical teaching. In a later paper, Irby^4^ focussed on what clinical teachers need to know in order to be effective educators. Essential clinical teacher characteristic included: “...knowledge of medicine, patients, context, learners, general principles of teaching and case-based teaching scripts.” A further important descriptor identified by Wlodkowski^5^ was that of the ‘motivating instructor’.

## Methods

In the 1990’s we investigated students’ and teachers’ perceptions of the characteristics of good clinical teachers using a Delphi system approach.^6^ This approach captures and interprets experts’ opinions about a topic being scrutinized via a structured communication and iterative process.^7^ The use of such a Delphi approach was crucial to identifying and quantifying clinical teacher competency. It allowed us to categorize the characteristics which teachers and students thought were necessary for good clinical teaching. In the initial phase of the Delphi process, we asked clinical teachers what they considered were the characteristics of a ‘competent’ clinical teacher. The results of this survey were used for clinical teacher development and presented at an Australasian medical education conference.^6^

## Results

The outcome measures identified four attributes which students and teachers (with some differences) felt to be critical for good clinical teaching:

1. **‘Clinically competent’**: Statements that characterized clinicians according to their knowledge base and professional attributes. Those professional attributes of clinicians were seen as important in order for them to be good role models in areas such as ethics, cultural sensitivity, reputation as skilled practitioners, and their ability to keep up to date. This domain therefore combined competence in technical knowledge and skills with personal characteristics which relate to responsibility.
2. **‘Efficient organizer’**: Observable characteristics that relate to efficiency, e.g., organization of material, time management, consistency, good concentration. This domain represents external behaviours that can be measured.
3. **‘Group communicator’**: These are general statements referring to the ability of the clinical teacher to communicate (and/or facilitate) effectively without recognising the individual directly, which often describe proficiency with group management. Statements related to these attributes describe expertise in global communication and possession of good social skills.
4. **‘Person-centred**: This aspect of teaching relates to the ability of the clinical teacher to recognise the needs of the individual (either patient or student). If the respondent mentioned that the teacher is interested in students or patients, or some similar personal statement (e.g., “empathises”, “sensitive”, “guided”, etc.), then that teacher can be categorized as being person-centred.

After this classification phase, we then established a set of questions to measure levels of difference or similarity between the clinical teachers’ and students’ perceptions. The responses showed us that the first three characteristics (clinically competent, efficient organizer and group communication) were more important to clinical teachers than to students. However, in the students opinion being person-centred was the most important characteristic.

Included in the surveys were statements (see Appendix) that were rated according to their levels of importance and ability to be taught, and each statement was aligned with one of the four characteristics cited above. To check for agreement in aligning these statements to the factors, Kappa statistics were computed to ensure inter-rater consistency across two raters and the results indicated that all agreement measures were highly significant (*p* < .01). Students were significantly more optimistic about the ‘teachability’ of the four characteristics in comparison with their teachers. Teachers apparently thought that they were unable to change their teaching behaviours which could indicate that teaching ‘styles’ are similar to entities that have fixed frames of reference, for example being teacher-centred, and therefore require a strong motivational component to enact change.^8^

## Conclusions

Expectations of success, and motivations, in changing teaching behaviour are influenced by the taught, declared and hidden curricula.^9^ The clinical teacher characteristics identified in this survey can be embedded in the taught and declared curricula. A possible explanation, however, for our finding that clinical teachers were less optimistic about improving their teaching skills could be linked to the pressures of the invisible or hidden curriculum.^10^ This highlights the challenges within medical education of synthesizing the three aspects of curricula.

The fact that students valued person-centred more than the other characteristics further supports the need for clinical teachers to not only be motivated to change but also to be student- and patient-centred. The importance of being student-centred has been well established in medical education;^11^ although the order at which it is placed within the priorities of teaching has not been so clearly established, students in our and other studies placed it high.

Although exceptional clinical teachers such as William Osler may be born, students regard the attributes of competent and good teachers as being ‘teachable’. Our studies and reflections suggest that there are identifiable characteristics of competent clinical teaching that can be used to inform systems of evaluation. It also appears that the total teaching environment, particularly the invisible curriculum, determines teachers’ perceptions of how capable they are of changing their own teaching approach. We suggest that Delphi surveys such as ours, where both students and teachers define their perceptions of competent clinical teaching, are a relatively non-threatening and effective mechanism to determine the need for teacher change in particular clinical environments, particularly as students are often more aware of the invisible curriculum than individual teachers. With the knowledge of these perceptions teachers are more likely to have confidence that behaviours may be changed to produce the ‘student-centred’ ideal. It is also acknowledged that later research has proposed a measure of clinical teaching effectiveness using a psychometrically sound instrument,^12^ which adds a further lens through which to examine this area of research.

## Appendix

The following statements were rated according to their level of importance and teachability using a Likert scale of 1[not at all] to 7 [always]:

1. Enthusiastic, keen, passion for chosen field, willing to teach
2. Gives a clear structure/system/method of examination, e.g., clear objectives
3. Demonstrates good clinical skills and provides practice
4. Encourages questions and participation in learning process
5. Enthusiastic about teaching and clinical medicine, energetic
6. Appropriately pitched content
7. Knowledgeable about subject area, well informed
8. Explains to students what they are doing as they go along
9. Does not put down questions/answers by students and make them feel stupid
10. Explains what is going on to patient
11. Good communication skills, e.g., legible and articulate, clear
12. Makes a determined effort to give us quality teaching
13. Organizes regular teaching sessions
14. Genuinely interested in subject area and in imparting knowledge/understanding.
15. Constructive, positively critical
16. Treat patients with dignity and creates goodwill
17. Encourages participation
18. Listens
19. Interested in teaching and selects interesting material
20. Ensures that students see wide variety of pathology/disease
21. Considerate of the patients whose illness is being used to teach the students
22. Humane especially with regard to patients
23. Relates to practical patient care by using clinical examples
24. Happy to have students sit in on consultations (patients willing)
25. Knowledgeable
26. Makes time for students, available
27. Prepared for the teaching session, e.g., timing, content, location etc
28. Interested/shows interest in teaching students
29. Connects and is up to date with literature and techniques
30. Distinguishes wood from the trees
31. Sensitive to needs and vulnerability of patients
32. Able to summarize key points
33. Develops rapport, good interaction skills
34. Introduces him/herself to patients and students
35. Explains things simply and clearly, “user friendly”
36. Good role model, e.g., non-judgemental, ethical
37. Discusses aspect of teaching prior to and/or after bedside contact
38. Good communication skills
39. Delivers appropriate feedback to students, well intentioned
40. An appreciation of the relevant points
